# Bacterial immunotherapy is highly effective in reducing recurrent upper respiratory tract infections in children: a prospective observational study

**DOI:** 10.1007/s00405-023-08035-4

**Published:** 2023-05-30

**Authors:** Laura Rebolledo, Carmen Rodríguez-Vigil, Luis Carmen, Eva Llorente, María Guallar, Jesús Villoria, Eugenio Vicente

**Affiliations:** 1 Department of Otorhinolaryngology, San Jorge Hospital, Avenida de Martínez de Velasco 36, 22004 Huesca, Spain; 2 School of Medicine, University of Zaragoza, Calle de Pedro Cerbuna 12, 50009 Saragossa, Spain; 3 Child and Adolescent Oncohematology Unit and Department of Pediatrics, Miguel Servet University Hospital, Paseo de Isabel La Católica 1-3, 50009 Saragossa, Spain; 4 Child Otorhinolaryngology Unit, Department of Otorhinolaryngology, Miguel Servet University Hospital, Paseo de Isabel La Católica 1-3, 50009 Saragossa, Spain; 5 Department of Design and Biometrics, Medicxact, S.L., Plaza de La Ermita 4, 28430 Alpedrete, Spain

**Keywords:** Absenteeism, Cohort studies, Immunity, Otolaryngology, Pediatrics, Vaccination

## Abstract

**Purpose:**

Whilst immunotherapy is an appealing option as it could reduce the burden of recurrent pediatric respiratory tract infections (RTI), there is limited evidence on its effectiveness and more research was requested in order to better understand this therapeutic modality.

**Methods:**

We performed a prospective cohort study involving 57 subjects to assess the safety and effectiveness a 3-month regimen of either typified or patient-specific bacterial lysates could have in reducing the number of RTIs in children aged 0 to 11 years with histories of recurrent episodes.

**Results:**

After a 6-month follow-up, the number of RTIs and school absenteeism dropped sharply and significantly, from an adjusted mean (standard error) of 0.6 (0.04) episodes/month to 0.1 (0.03) episodes/month (74.7% reduction, *P* < 0.001), and from an adjusted mean score of 4.6 (1.06) points to 0.0 (0.01) points over 10 (99.5% reduction, *P* < 0.001), respectively. There was also a significant decrease in the severity of symptoms. No adverse reactions were observed.

**Conclusion:**

The use of the study product is associated with a decreased risk of recurrent RTIs in children, with a very favorable safety profile that warrants further investigation in randomized clinical trials.

**Supplementary Information:**

The online version contains supplementary material available at 10.1007/s00405-023-08035-4.

## Introduction

Children are particularly susceptible to ear, nose and throat (ENT) infections due to the relative immaturity of their immune systems and increased pathogenic microbial exposure [1, 2]. They often have a recurring pattern, characterized by a vicious cycle of defective immune responses, infection, and mucosal inflammation that further heightens immune defects [3]. Their incidence can be high, affecting up to 25% of children younger than one year and 18% of those 1 to 4 years old [2]. Recurrent ENT infections have been associated with antibiotic misuse [4], a considerable economic burden [5] and the emergence of complications such as severe infections of the lower respiratory tract, the development of chronic conditions or the need for surgical treatment [1]. Understandably, research has focused on unveiling treatments to reduce the number of episodes through the stimulation of immune responses [2]. Non-specific immunostimulation or immunomodulation of innate immune responses by means of vaccine-immunostimulant combinations is one such strategy [6, 7].

The protection of the respiratory tract features a mechanical barrier composed of a ciliated epithelium coated with mucous enriched with proteins exerting antibacterial, immunomodulating and protective functions driven by the so-called mucosa-associated lymphoid tissues that develop gradually during infancy [2, 8]. In particular, there is a relative bias away from toll-like receptors (TLR) 1 to 7 and T helper 1-cell-type (T_H_1) responses in the neonatal respiratory tract that requires repeated low-dose exposure to environmental and commensal TLR agonists in order to mature [9, 10]. In consonance with the three-decade old proposition of the hygiene hypothesis [11] and the microbiome depletion hypothesis [12], reduced exposure to microbial products and, interestingly enough, inadequate antibiotic use, would prevent maturation (polarization towards T_H_1 at the expense of T_H_2, among others) of the infant immune response. This would boost both repeated infections as well as autoimmune and allergic disorders, which are on the rise in developed countries [12].

It has been proposed that the biopharmaceutical development of molecules that modulate innate immunity may be of clinical relevance in pediatric medicine [9]. Immunostimulants prepared from lyophilized bacterial extracts have been observed to activate mucosal dendritic cells, macrophages, CD4^+^ helper, CD8^+^ cytotoxic and natural killer T lymphocytes by pattern recognition receptors (such as TLRs)-dependent signaling. In addition, these immunostimulants prime naive lymphocytes and enhance IgA responses at mucosal surfaces [7, 9, 13]. Importantly, the activation of such innate effector mechanisms could up-regulate T_H_1 responses and offer preventive and therapeutic efficacy against both bacterial and viral infections throughout anatomically related mucosal sites [8, 14, 15]. Several reviews or meta-analyses have consistently shown that these therapies reduce the incidence of respiratory tract infections in childhood [16-21], and are authorized for such indication by the European Medicines Agency [22]. Although we do not know which regulatory immune pathways specifically train the immune system, an empirically effective intervention to reduce recurrent infections in susceptible children and consequently lower antibiotic consumption, may be an important step in curtailing the associated burden and the occurrence of antibiotic resistance.

The present report concerns an observational cohort study of bacterial vaccines-immunostimulants on children suffering from recurrent acute episodes of otitis media (middle ear infections) (AOM) or pharyngotonsillitis (PT). The study products were vaccine-immunostimulant combinations composed of a suspension of bacterial subcellular fractions obtained from bacterial lysate of either typified strains (specific vaccines) or cultures of the patients’ own exudates (auto-vaccines). We aimed to assess their effectiveness in lowering the incidence and severity of repeated respiratory tract infections as well as their impact on school absenteeism and some immunologic markers.

## Materials and methods

### Study design

This was a non-interventional prospective cohort, investigator-initiated study. The clinical investigators, who are the authors of this article, designed and conducted it at a hospital in Zaragoza, Spain. Probelte Pharma, S.L.U. provided funds for logistical aspects. The Ethics Committee of the Miguel Servet Hospital approved the study protocol prior to start. This research was performed in accordance with the principles of the Declaration of Helsinki.

### Selection of patients

Subjects under 16 years of age referred for the first time to the Child Otorhinolaryngology Unit of the study site for the assessment and treatment of recurrent AOM or PT episodes were eligible for enrollment. Patients could be included if they met our routine criteria for starting sublingual immunotherapy, namely: having had four or more AOM episodes in the previous year [23] (AOM subgroup) or recurrent PT meeting the criteria for tonsillectomy of the Spanish ENT Society (PT subgroup) [24]. Children with primary immunologic deficiency syndromes, except for isolated IgA deficiency, certain diseases that could interfere with the assessments of the study (Table S1 in the supplementary material), or suspected hypersensitivity to the study products were excluded. All participants and their representatives provided written informed consent.

### Exposition of interest

**Fig. 1:**
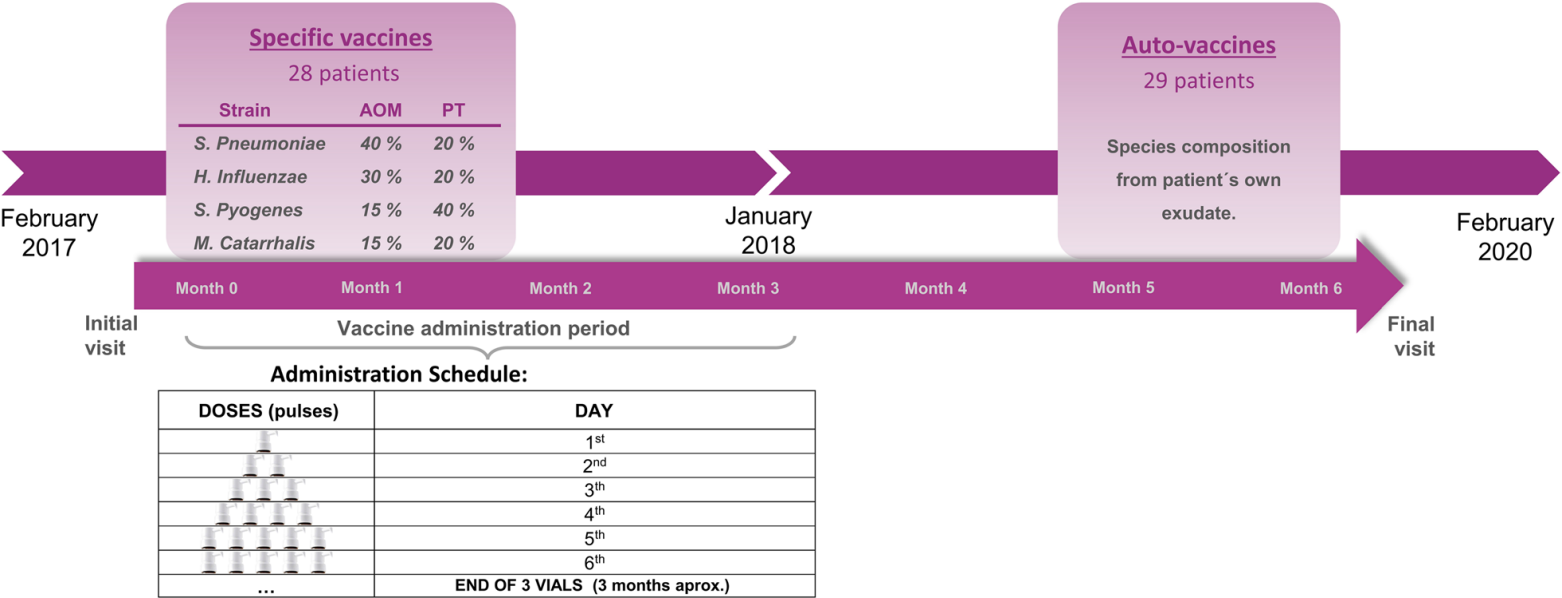
Design of the study

Subjects who met the selection criteria and were referred to the Unit were included consecutively. Specific vaccines (Probelte Pharma) were used during the first part of the study, from February 2017 to January 2018. From then on, auto-vaccines (Probelte Pharma) were introduced up to the end of recruitment in February 2020 (Fig. 1). For the specific vaccines, precise strains of *S. pneumoniae*, *H. influenzae*, *S. pyogenes* and *M. catarrhalis* were cultured separately, killed and lysed. Each extract was then purified and mixed in 40/30/15/15% volume proportions for AOM subgroup and 20/20/40/20% volume proportions for PT subgroup (Fig. 1). The manufacturing process was similar for the auto-vaccines, except that the starting species were those isolated from the patients’ own exudates (see Table 1 for details on the composition of the vaccines). The final product was in the form of sublingual solution, containing 2,000 million germs per milliliter. No adjuvants were added.

**Table 1:** Composition of the vaccines

	Specific vaccines^a^	Auto-vaccines^b^	
	AOM (*N* = 17)	PT (*N* = 11)	*N* = 29
*S pneumoniae*	40.0	20.0	–
*H. influenzae*	30.0	20.0	–
*S. pyogenes*	15.0	40.0	–
*M. catarrhalis*	15.0	20.0	10.0
*K. pneumoniae*	–	–	10.0
*Streptococcus* spp.	–	–	52.0
*Staphylococcus* spp.	–	–	38.0
*Corynebacterium* sp.	–	–	17.0
*Pseudomonas* sp.	–	–	14.0

*The available number of cases was used in the denominators of the relative frequencies*

^a^The figures are the volume percentages of specified species within the finished vaccine product (add to 100%)

^b^The figures are percentage of vaccines containing each specified species (do not add to 100%)

### Study procedures

The subjects received three vials containing seven milliliters of the solution featuring a device that released a stable amount of content. The administration was started gradually, with one, two, three, four and five sublingual pulses on the first, second, third, fourth and fifth treatment days, respectively. Thereafter, five pulses were administered every day until all the three vials were used (approximately three months) (Fig. 1).

Subjects were seen at enrolment and after six months. The initial visit included a brief medical history on respiratory tract infections and a physical examination. An otological and tonsillar ring examination was performed on both study visits. Information about treatments administered for the AOM or PT episodes, as applicable, was also collected. Blood samples for a basic hematology, the determination of lymphocytic populations, and concentrations of immunoglobulins, anti-*S. pneumoniae* capsular polysaccharide and tetanus toxin antibodies, tumor necrosis factor (TNF) alpha, and interleukin (IL) 1, 6 and 8 were taken at both visits as well. The local facilities were used for all determinations except anti-*S. pneumoniae* and tetanus toxin antibodies, TNF alpha, and ILs, which were done in a central laboratory.

### Outcomes

The number of infectious episodes (AOM or PT) suffered during the previous year and since the baseline assessment were collected retrospectively at the initial and follow-up visits, respectively. Parents were given a short form to annotate the occurrence of episodes between study visits. We also tried to retrieve reliable information on past infectious episodes through electronic medical records, but we had to rely on parents’ and patients’ verbal statements and filled forms when the electronic records were unavailable. The primary outcome was the incidence rate of infectious episodes per month, calculated from the total number of episodes during the recalling periods. Secondary outcomes included the severity of AOM/PT symptoms and the degree of school absenteeism, both measured by visual analogue scales (VAS) of 100 mm in length, and the results of clinical and laboratory immunological examinations. Since we believed that the more severe the episodes, the more imprint they left on patients, we asked them to pay special attention to the worst recalled episode when there were more than one in order to reduce recall bias.

### Statistical analysis

Appropriate descriptive statistics were calculated by cohort (specific vaccines and auto-vaccines) and for the whole study sample. Separate descriptions were also prepared for the AOM and PT subgroups. Bi-variable pre-post comparisons within cohorts were carried out either with paired *t*/Wilcoxon signed rank or using McNemar’s tests, whereas comparisons between cohorts were done with either *t*/Mann–Whitney’s *U* or Fisher’s exact/Pearson’s chi-squared tests, as appropriate. The analysis of the primary outcome was done using a generalized linear mixed model of the mean number of episodes per child per month with random intercepts for study subjects using the Poisson distribution for the errors. The fixed predictors were study visit, type of vaccine, categorized baseline inter-episode interval (lower/equal or greater than one month), and their interactions with study visit. Differences in follow-up durations (one year and six months for the initial and follow-up visits, respectively) were accounted for by adjusting the offset parameter. Similar models were used to analyze the severity of symptoms and school absenteeism scores, but using the Gamma distribution for the errors and a (non-canonical) logarithmic link. These models also included etiology (OMA or PT), history of bronchitis (only for the model of the severity of symptoms), IgG_2_ concentration and their interactions with study visits as fixed predictors. The retention of fixed predictors was guided by Wald tests of nullity of coefficients, keeping those getting P values less than 0.25 for either the linear terms or their interactions with study visits. Only observed data was used in the main analysis; missing data was not imputed. However, as about 40% of the intensity and absenteeism scores were missing at the six-month follow-up, sensitivity analyses were performed after conservatively calculating and imputing them by their baseline values adjusted according to the reduction of the incidence of infectious episodes (note that this denies any direct effect of treatment, other than those mediated by the incidence of episodes).

The sample size was calculated at 51 patients to achieve 80% power at a 0.05 significance level to detect that a baseline rate of 0.6 infectious episodes per month is reduced by half in a single Poisson regression model over a mean exposure time of six months. Two-sided P values of 0.05 or less were considered to indicate statistical significance. The version 9.4 of the SAS system (Cary, NC, USA) was used in all analyses.

## Results

### Characteristics of the patients

**Fig. 2:**
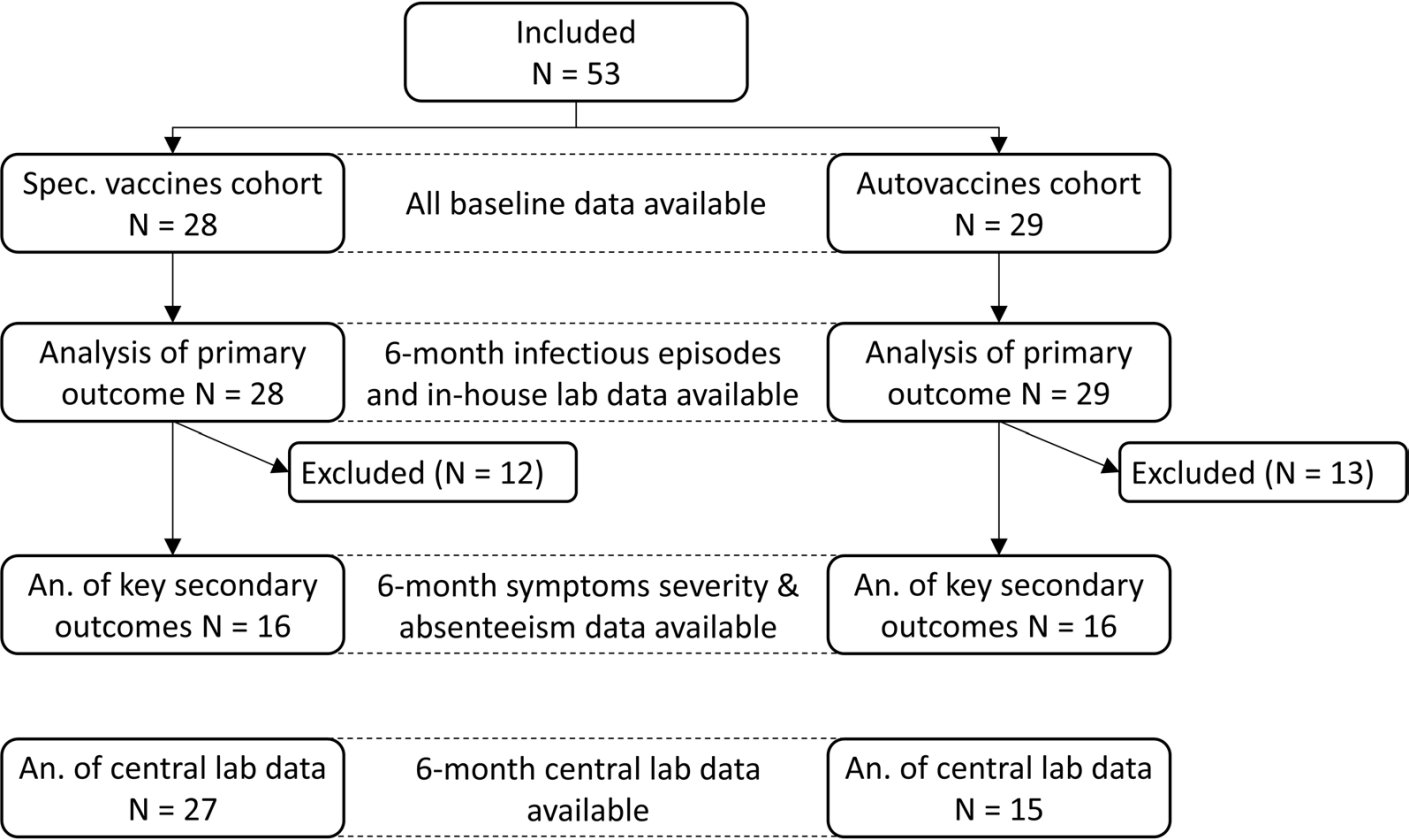
STROBE flow diagram

A total of 57 patients were enrolled; 28 patients were treated with the specific vaccines, and 29 patients were treated with the auto-vaccines. Six-month data regarding the number of infectious episodes and information from in-house clinical laboratory examinations was available from all these patients. The 6-month scores on symptom severity and absenteeism were only available from 16 patients from each cohort, and the 6-month data from the central laboratory were available from 27 patients in the specific vaccines cohort and 15 patients in the auto-vaccines cohort (Fig. 2).

The median age and the proportion of males were 60.5 months and 60.7% in the specific vaccines cohort, and 59.0 months and 37.9% in the auto-vaccines cohort. In these respective cohorts, 57.1% and 27.6% of patients had mainly AOM episodes (AOM subgroup, formed by 24 patients), whilst 42.9% and 72.4% of patients had mainly PT episodes (PT subgroup, formed by 33 patients). Although the distribution of the predominant etiologies (AOM or PT) was uneven (Pearson’s chi-square *P* = 0.047), the differences of other socio-demographic or clinical characteristics between the study cohorts did not reach statistical significance, with the exception of the score of school absenteeism, which was higher in the auto-vaccines cohort (Mann–Whitney’s *U* test *P* = 0.004, Table 2). The median number of infectious episodes during the previous year did not differ significantly between the specific vaccines cohort (6.0) and the auto-vaccines cohort (7.0).

**Table 2:** Clinical features of patients at baseline

	Specific vaccines	Auto-vaccines
	*N* = 28	*N* = 29
Median age (IQR)—years	4.5 (2.5–6.0)	4.0 (3.0–6.0)
Median age (IQR)—months	60.5 (34.0–81.5)	59.0 (36.0–75.0)
Median gestational age at birth (IQR)—weeks	39.0 (36.0–40.0)	39.0 (38.0–41.0)
Male sex—no. (%)	17 (60.7)	11 (37.9)
Prior schooling—no. (%)	26 (96.3)	27 (100.0)
History of bronchitis—no. (%)	9 (32.1)	15 (51.7)
History of pneumonia—no. (%)	7 (25.0)	5 (17.2)
History of any lower respiratory tract infection—no. (%)	13 (46.4)	18 (62.1)
Etiology—no. (%) ^a^		
Predominant AOM	16 (57.1)	8 (27.6)
Predominant PT	12 (42.9)	21 (72.4)
Baseline inter-episode interval—no. (%)		
Less than or equal to one month	17 (70.8)	13 (56.5)
Between one and three months	6 (25.0)	10 (43.5)
Greater than three months	1 (4.2)	0 (0.0)
Baseline therapies—no. (%)		
Antimicrobials	28 (100.0)	29 (100.0)
Anti-inflammatories	27 (96.4)	29 (100.0)
Mucolytic	2 (7.1)	0 (0.0)
Antitussives	1 (3.6)	0 (0.0)
Corticosteroids	6 (21.4)	2 (6.9)
Otological examination—no. (%) ^b^		
Normal	8 (50.0)	4 (50.0)
Suppurative otitis media (middle ear infection)	6 (37.5)	1 (12.5)
Tympanic perforation	2 (12.5)	3 (37.5)
Tonsil grades—no. (%)^ c,d^		
I	2 (16.7)	0 (0.0)
II	3 (25.0)	9 (42.9)
III	6 (50.0)	12 (57.1)
IV	1 (8.3)	0 (0.0)
Median episodes in the previous year (IQR)—no	6.0 (5.0–10.0)	7.0 (6.0–10.0)
Median int. of symptoms in the prev. year (IQR)—VAS score ^e^	7.2 (5.3–8.0)	7.0 (5.0–8.0)
Median absenteeism in the prev. year (IQR)—VAS score ^f,^^g^	4.0 (1.0–6.8)	7.0 (5.0–8.0)

*int.* intensity, *IQR* interquartile range, *no.* number, *prev.* previous, *VAS* visual analogue scale. The available number of cases was used in the denominators of the relative frequencies

^a^The differences between study cohorts were statistically significant (Pearson’s chi-squared test *P* = 0.047)

^b^Described for the 24 subjects who had mainly recurrent AOM episodes

^c^According to the 4-grade classic staging system of Brodsky et al. [48]

^d^Described for the 33 subjects who had mainly recurrent PT episodes

^e^The score ranges between 0 (no symptoms) to 10 (maximum imaginable intensity)

^f^The score ranges between 0 (no absenteeism) to 10 (maximum absenteeism—does not attend school at all)

^g^The differences between study cohorts were statistically significant (Mann–Whitney’s *U* test *P* = 0.004)

**Fig. 3:**
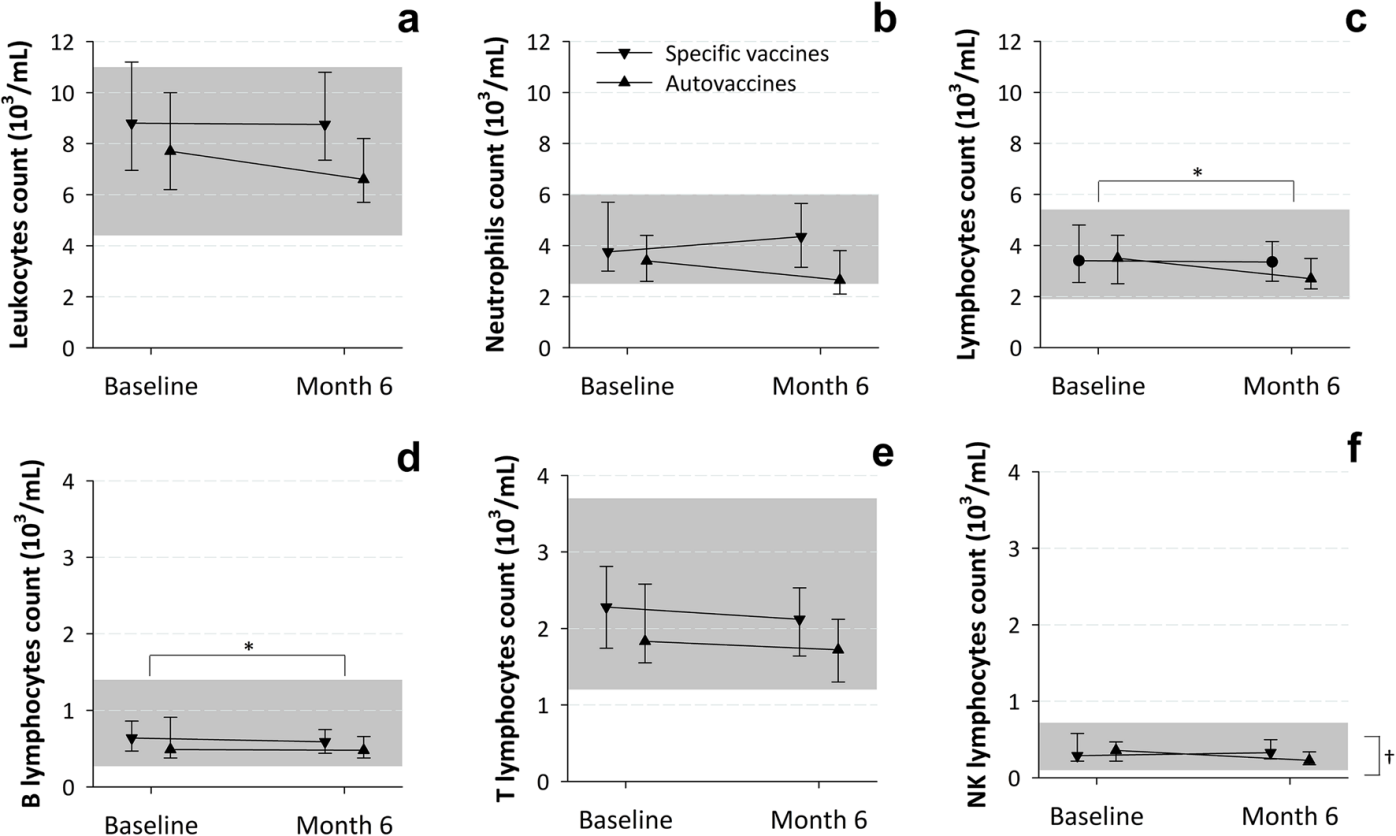
Evolution of lab parameters (leukocytes, neutrophils and total, T, B and NK lymphocytes count) by study cohort. *mL* milliliter. Values are medians and interquartile ranges. The gray rectangles indicate the normal ranges (taken from [28]). *The within-group changes from baseline were statistically significant. ^†^The between-group changes from baseline were statistically significant

**Fig. 4:**
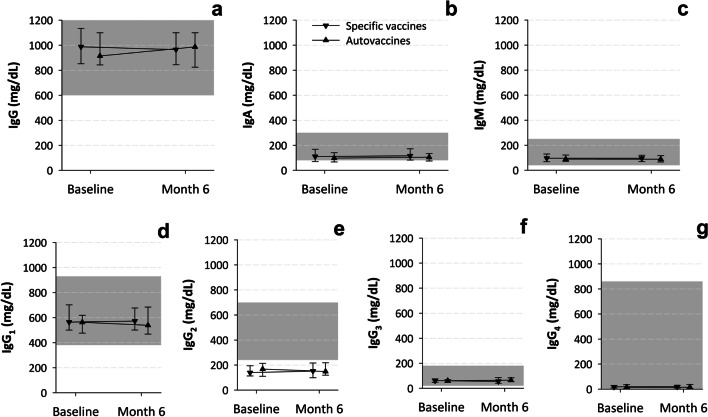
Evolution of lab parameters (immunoglobulin concentration) by study cohort. *dL* deciliter, *Ig* immunoglobulin, *mg* milligram. Values are medians and interquartile ranges. The gray rectangles indicate the normal ranges (taken from [26])

**Fig. 5:**
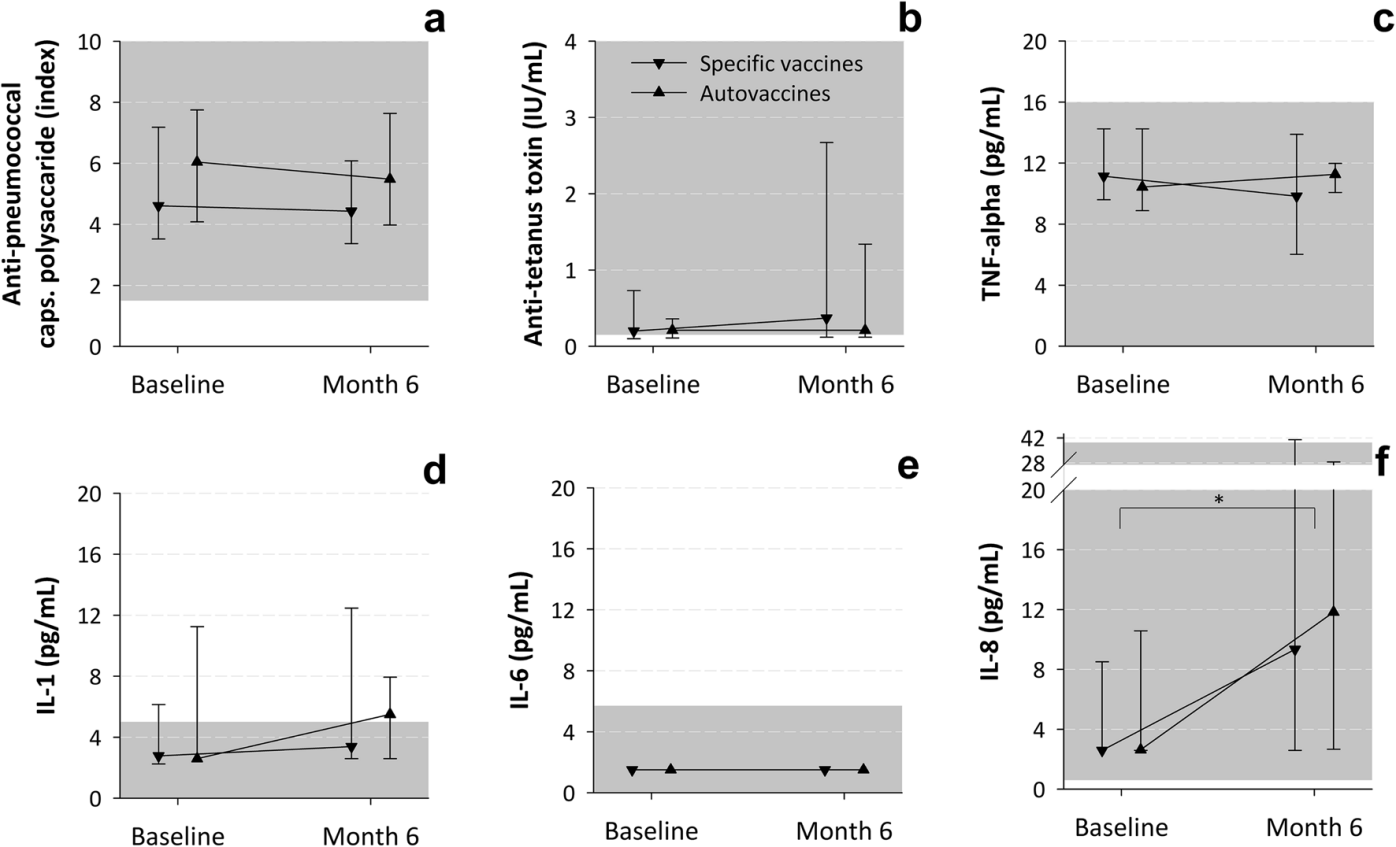
Evolution of lab parameters (anti-pneumococcal capsular polysaccharide, anti-tetanus toxin, TNF-alpha and interleukin levels) by study cohort. *caps.* Capsular,* IU* international units, *IL *interleukin, *mL* milliliter, *pg* picogram, *TNF* tumor necrosis factor. Values are medians and interquartile ranges. The gray rectangles indicate the normal ranges (taken from [25, 27, 29]). *The within-group changes from baseline were statistically significant

Neither were there statistically significant differences between the study cohorts in the values of the laboratory parameters (Table 3 and Figs. 3, 4, 5), which were within normal ranges (see the references cited in the footnotes of Figs. 3, 4, 5).

**Table 3:** Clinical laboratory parameters at baseline

	Specific vaccines	Auto-vaccines
	*N* = 28	*N* = 29
Median leucocytes (IQR)—cells × 10^3^/μL	8.8 (7.0–11.2)	7.7 (6.2–10.0)
Median neutrophils (IQR)—cells × 10^3^/μL	3.8 (3.0–5.7)	3.4 (2.6–4.4)
Median neutrophils (IQR)—% within leucocytes	48.9 (40.9–53.7)	44.0 (39.1–52.8)
Median lymphocytes (IQR)—cells × 10^3^/μL	3.4 (2.6–4.8)	3.5 (2.5–4.4)
Median lymphocytes (IQR)—% within leucocytes	39.3 (33.6–48.7)	42.1 (36.2–49.8)
Median B lymphocytes (IQR)—cells × 10^3^/μL	0.6 (0.5–0.9)	0.5 (0.4–0.9)
Median B lymphocytes (IQR)—% within lymphocytes	19.9 (16.2–23.5)	18.5 (14.6–23.7)
Median T lymphocytes (IQR)—cells × 10^3^/μL	2.3 (1.7–2.8)	1.8 (1.6–2.6)
Median T lymphocytes (IQR)—% within lymphocytes	66.5 (62.3–69.8)	66.6 (62.7–71.9)
Median NK lymphocytes (IQR)—cells × 10^3^/μL	0.3 (0.2–0.6)	0.4 (0.2–0.5)
Median NK lymphocytes (IQR)–% within lymphocytes	10.2 (8.1–14.0)	11.4 (8.0–15.8)
Median IgG (IQR)—mg/dL	988 (852–1135)	914 (843–1100)
Median IgG_1_ (IQR)—mg/dL	565 (501–702)	563 (477–619)
Median IgG_2_ (IQR)—mg/dL	139 (113–195)	168 (110–214)
Median IgG_3_ (IQR)—mg/dL	61 (48–75)	60 (47–69)
Median IgG_4_ (IQR)—mg/dL	18 (9–30)	14 (5–35)
Median IgA (IQR)—mg/dL	111 (70–168)	98 (67–141)
Median IgM (IQR)—mg/dL	98 (68–130)	89 (72–122)
Median anti-caps-PS IgG (IQR)—protection index ^a^	4.6 (3.5–7.2)	6.0 (4.1–7.8)
Median anti-tetanus toxin IgG (IQR)—IU/mL	0.2 (0.1–0.7)	0.2 (0.1–0.4)
Median TNF-alpha (IQR)—pg/mL	11.1 (9.6–14.2)	10.4 (8.9–14.2)
Median IL-1 (IQR)—pg/mL	2.8 (2.3–6.1)	2.6 (2.6–11.3)
Median IL-6 (IQR)—pg/mL ^b^	1.5 (1.5–1.5)	1.5 (1.5–1.5)
Median IL-8 (IQR)—pg/mL	2.6 (2.6–8.5)	2.6 (2.6–10.6)

*caps-PS.* *S. pneumoniae* (pneumococcal) capsular polysaccharide, *dL* deciliter, *Ig* immunoglobulin, *IL* interleukin, *IQR* interquartile range, *IU* international units, *mL* milliliter, *μL* microliter, *no.* number, *pg* picogram, *TNF* tumor necrosis factor

^a^Number of times the sample activity is greater than the 50% killing baseline activity; 1 denotes the minimum level of protection

^b^The result was below the detection threshold of 1.5 pg/mL in 81 of 97 samples analyzed (83.5%), which were assigned this value

### Primary endpoint

**Fig. 6:**
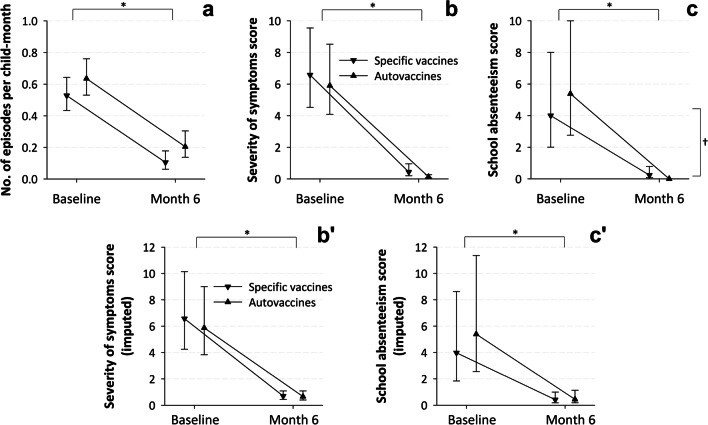
Evolution of the adjusted incidence rates of infectious episodes (panel **A**), severity of AOM/PT symptoms (without and with imputation, panels **B** and **B**’) and school absenteeism scores (without and with imputation, panels **C** and **C**’) by study cohort. Values are least square means and 95% confidence intervals from the generalized linear mixed models back-transformed to response scale. *The within-group changes from baseline were statistically significant. ^†^The between-group changes from baseline were statistically significant

The immunotherapy was associated with a 74.7% (95% confidence interval [CI], 58.5% to 84.6%) decrease in the incidence of infectious episodes. The adjusted mean (standard error) incidence rate fell from 0.6 (0.04) episodes per month-child at baseline to 0.1 (0.03) episodes per month-child at the 6-month follow-up. Within the specific vaccines and auto-vaccines cohorts, the respective figures were 0.5 (0.05) and 0.6 (0.06) episodes per month-child at baseline and 0.1 (0.03) and 0.2 (0.04) episodes per month-child at follow-up (Fig. 6A). The change from baseline was statistically significant (*P* < 0.001), but there were no significant differences between study cohorts (*P* = 0.130) (Table S2 in the supplementary material). The baseline inter-episode interval duration was inversely associated with the incidence of infectious episodes at both study assessments (intuitively, longer intervals correspond to less episodes).

### Secondary effectiveness endpoints

The immunotherapy was also associated with a 96.1% (95% CI 91.5 to 98.2%) decrease in the severity of symptoms. The adjusted mean score fell from 6.2 (0.76) points at baseline to 0.2 (0.06) points over 10 at the 6-month follow-up. Within the specific vaccines and auto-vaccines cohorts, the figures were 6.6 (1.16) and 5.9 (1.02) points at baseline, and 0.4 (0.16) and 0.1 (0.04) points at follow-up, respectively (Fig. 6B). The change from baseline was statistically significant (*P* < 0.001), but there were no significant differences between study cohorts (*P* = 0.073) (Table S2 in the supplementary material). The shorter baseline inter-episode intervals in the PT subgroup and the lower baseline IgG_2_ levels in both subgroups were significantly associated with higher severity scores at both study assessments. History of bronchitis was associated with greater severity reductions.

School absenteeism also decreased by 99.5% (95% CI, 98.6% to 99.9%). The adjusted mean score fell from 4.6 (1.06) points at baseline to 0.0 (0.01) points over 10 at the 6-month follow-up. Within the specific vaccines and auto-vaccines cohorts, the respective figures were 4.0 (1.31) and 5.4 (1.71) points at baseline, and 0.2 (0.13) and 0.0 (0.00) points at follow-up (Fig. 6C). The change from baseline was statistically significant (*P* = 0.001), and was also significantly higher in the auto-vaccines cohort than in the specific vaccines cohort (*P* < 0.001) (Table S2 in the supplementary material). Shorter baseline inter-episode intervals and lower baseline IgG_2_ levels were significantly associated with smaller and larger absenteeism reductions from baseline, respectively. Both were also associated with higher absenteeism scores in general.

The laboratory immunological examinations were in general within normal ranges [25-29] at both visits and showed little variation from baseline (Figs. 3, 4, 5). The exception was IgG_2_ concentration, which was below normal range. Total- and B-lymphocyte counts decreased significantly (Fig. 3C, D) and IL-8 concentration increased significantly (Fig. 5F) between study assessments. NK-lymphocyte counts decreased in the auto-vaccines subgroup and remained unchanged in the specific vaccines subgroup; this difference reached statistical significance (Fig. 3F).

The sensitivity analyses that used a worst-case scenario for missing symptom intensity and absenteeism scores at follow-up yielded very similar results (Fig. 6B’ and C’).

### Safety

The vaccines were very well tolerated. All patients completed the 3-month immunization cycle provided for in the protocol and no relevant adverse reactions were reported.

## Discussion

In this observational study we have evidenced a remarkable reduction in the frequency, intensity and consequences of AOM and PT episodes after six months in a cohort of children with repeated infections who underwent a three-month treatment with bacterial vaccine-immunostimulant combinations prepared from bacterial lysates either from typified species (polyvalent specific vaccines) or from species isolated from patients’ own exudates (auto-vaccines).

Prevention is widely recognized as the most important intervention in reducing the disease burden associated with repeated respiratory tract infections in children. Since the elimination of environmental triggering factors is unfeasible, immunotherapy is considered the most effective preventative strategy [30]. Our results suggest that the bacterial preparations assessed in the present study were highly effective for this purpose, which could mean significant reductions of the associated clinical [1, 31] and financial [5] burdens, antibiotic prescription and resistance [32, 33], and medical complications [2, 34]. Noteworthy, we also found a clear reduction in school absenteeism, which was traditionally elusive in previous related studies [7]. In turn, deterrence of microbial resistance is of great importance, since it is regarded as one of the current major health problems worldwide [35].

Despite the attractiveness of immunotherapy for the prevention of recurrent ENT infections in children, there is only a sufficient body of randomized trials for a single particular bacterial lysate product [36]. Most studies have shown it to be protective against recurrent upper respiratory tract infections [6, 16, 18, 39,37-] despite some conflicting reports [40], which could be partially explained by the various methods by which the lysates were prepared [41]. Notwithstanding this considerable amount of favorable evidence concerning the use of this product in upper respiratory tract infections, it is still seldom recommended for ENT infections and in general is not shared among clinicians [30]. The promising results in our study support the broader recommendation of immunotherapy for the prevention of ENT infections.

The magnitude of the changes from baseline in this study is larger than the between group differences reported for similar outcomes in previous placebo-controlled clinical studies [6, 16, 18, 37]. This suggests that there were benefits other than those of the intervention that should be accounted for by the use of a placebo control. In our opinion, this is the main threat to the robustness of our results, which would remain somewhat uncertain until they are confirmed in an interventional setting. However, it is known that mucosal delivery can produce greater responses than subcutaneous delivery, probably related to a comparatively higher efficiency of dendritic cells than macrophages for antigen processing [42]. A similar phenomenon might apply in relation to oral (digestive) delivery, which was the most widely used administration route in the reviews cited above.

We did not find an association between the baseline frequency of infectious episodes and the effectiveness of the intervention, as shown with other bacterial vaccine-immunostimulant preparations [16, 43]. The reduced sample size and the categorization of this information might have precluded us from finding significant associations.

The interaction of the immunostimulant product with the TLRs on the surface of mucosa-associated dendritic cells would lead to their activation through mitogen-associated protein kinase signaling and nuclear factor-κB translocation and activation, migration to lymph nodes and secretion of transforming growth factor β, interferon alpha and IL-6 [7, 44]. These cytokines can favor the recruitment of innate effector cells to epithelial surfaces and can also promote the lymphocyte function, induce the secretion of other pro-inflammatory interleukins and TNF-α, and activate macrophages and NK cells [13, 15, 44]. In this setting, priming of T lymphocytes would also occur, which in turn would modulate B-cell isotype switching for IgA production [7]. As a net result, the vaccine-immunostimulant preparations would provide direct protection by amplifying natural defenses and reducing respiratory tract inflammation induced by infections and allergens [43]. Indirect protection could result from changes in the normal nasopharyngeal flora [45]. This is linked to reduction of carriage, antibiotic resistance and spreading to siblings, enhanced responses against viral infection or coinfection, as well as to the replacement of vaccine-type with non-vaccine-type serotypes [15, 45].

Tracking these mechanisms may, however, be less immediate than it seems at first glance. The changes in laboratory parameters, including some immunologic markers, were small or negligible in the present study, with the exception of the increase in IL-8 concentration. Delayed evaluation of laboratory parameters after the completion of the treatment cycle may have reduced the likelihood of tracing subtle or short-lived variations of immune parameters, although in a prospective cohort study that searched for markers of both humoral and cellular immune mechanisms just after finishing immunization with polyvalent bacterial preparations in respiratory tract infection-prone subjects, only an increase of the proliferative capacity of antigen-specific memory CD3^+^/CD4^+^ T lymphocytes could be observed; the levels of total immunoglobulins, specific antibodies or T-, B-, and NK-cells subsets remained unchanged [15]. Since the major functions of IL-8 relate to chemotaxis for neutrophils and T-lymphocytes [46], the observed increase might have to do with the aforesaid immune events following the activation of mucosal dendritic cells, and suggests that the study product targets immunostimulatory, rather than regulatory cells, which is a major determinant of mucosal vaccine formulations [8].

The most obvious limitations of this study are its observational nature and the lack of a comparative control group to discern the placebo effect. Despite the unquestionable benefit of a near eradication of infectious episodes coinciding with immunotherapy, it is unclear to what extent this responded to a cause-effect relationship. The lack of carefully controlled trials on immunotherapies for repeated pediatric ENT infections has long been stated [47]. As a consequence of the above, a well-designed randomized placebo-controlled trial of the study product is warranted by current results. We did not measure healthcare costs, antibiotic days or morbidity; nod did we evaluate the serotypes present in the nasopharynx before and after the intervention to assess the variation of carriage and eventual replacement phenomena in the nasopharyngeal flora. These data could have permitted greater insight into the clinical and biological effects of immunotherapy. Retrospective information of infectious episodes is recall bias-prone, particularly when the medical records were not available to contrast patients’ declarations. Nonetheless, we deem that recall bias is unlikely when no episodes were reported at all, as was frequently the case. In addition, for the reasons stated in the methods section, we focused on the most severe episode when more than one was reported to reduce inaccurate recall of symptom severity. Due to the limited sample size, we were also unable to assess the effects on sensitive subgroups such as children with chronic serous otitis or suppurative complications of PT. Notwithstanding, we were able to evaluate as many patients as planned and thus, we had enough statistical power to address the primary aim of detecting a clinically relevant reduction in the incidence of infectious episodes.

In conclusion, this research suggests that the sublingual lysates evaluated are safe and effective enough to offer protection against ENT recurring infections in infection-prone children. This warrants and may guide the design of a randomized placebo-controlled clinical trial that would not only provide robust evidence to complement the thus far limited body of well-conducted research with these therapies, but would also delve into mechanisms of action through, for example, nasopharynx sampling to assess population and phenotypic changes of immunocytes. The advantage of auto-vaccines over specific vaccines in reducing school absenteeism should also be further explored.

## Supplementary Information

Below is the link to the electronic supplementary material.

Supplementary file1 (DOCX 44 KB)

## Data Availability

Dr. Eugenio Vicente and Dr. Laura Rebolledo, from the Department of Otorhinolaryngology of the Miguel Servet University hospital and San Jorge hospital, respectively, will oversee the dataset. Granting access to this information will be evaluated on a case-by-case basis, upon reasonable request by the interested party. Data access requests should be addressed to Dr. Eugenio Vicente at eavicente@gmail.com.
