# Supplementary material for: Bacterial immunotherapy is highly effective in reducing recurrent upper respiratory tract infections in children: a prospective observational study
Source: Eur Arch Otorhinolaryngol. 2023 May 30;280(10):4519–30. doi: 10.1007/s00405-023-08035-4 (PMC10477114; doi:10.1007/s00405-023-08035-4)
Supplement: Supplementary file 1 — Supplementary file1 (DOCX 44 KB) [file 405_2023_8035_MOESM1_ESM.docx]

**Supplementary Information**

**Article Title:** Bacterial immunotherapy is highly effective in reducing recurrent upper respiratory tract infections in children: A prospective observational study

**Author Information**

Laura Rebolledo, MD (0000‑0003‑1169‑6499),^1^ Carmen Rodríguez‑Vigil, MD PhD (0000‑0002‑9377‑2610),^2^ Luis Carmen, MD (0000‑0001‑7518‑2257),^3^ Eva Llorente, MD (0000‑0002‑4282‑2139),^3^ María Guallar, MD (0000‑0002‑5911‑1667),^3^ Jesús Villoria, MD BStat (0000‑0001‑6508‑9083),^4^ Eugenio Vicente, MD PhD (0000‑0001‑6724‑5158)^5^

^1^ Department of Otorhinolaryngology, San Jorge Hospital, Avenida de Martínez de Velasco 36, 22004 Huesca, Spain, and School of Medicine, University of Zaragoza, Calle de Pedro Cerbuna 12, 50009, Zaragoza, Spain

^2^ Child and Adolescent Oncohematology Unit and Department of Pediatrics, Miguel Servet University Hospital, Paseo de Isabel la Católica 1‑3, 50009 Zaragoza, Spain

^3^ Child Otorhinolaryngology Unit, Department of Otorhinolaryngology, Miguel Servet University Hospital, Paseo de Isabel la Católica 1‑3, 50009 Zaragoza, Spain

^4^ Department of Design and Biometrics, Medicxact, S.L., Plaza de la Ermita 4, 28430 Alpedrete, Spain

^5^ Child Otorhinolaryngology Unit, Department of Otorhinolaryngology, Miguel Servet University Hospital, Paseo de Isabel la Católica 1‑3, 50009 Zaragoza, Spain

Correspondence to Dr. Eugenio Vicente. Department of Otorhinolaryngology, Miguel Servet University Hospital, Paseo de Isabel la Católica 1‑3, 50009 Zaragoza, Spain, and School of Medicine, University of Zaragoza, Calle de Pedro Cerbuna 12, 50009, Zaragoza, Spain. Tel.: +34.976.765.500. E‑mail: eavicenteg@gmail.com.

**Table of Contents**

[Table S1: Selection criteria 2](#_Toc127429812)

[Table S2: Solutions of the generalized linear mixed models 3](#_Toc127429813)

**Supplementary Tables**

# Table S1: Selection criteria

| Inclusion criteria: |
| --- |
| 1. Patients aged between 0 and 16 years. |
| 2. Consecutive patients referred for the first time to the investigator’s practice because of recurrent ENT infections. |
| 3. Patients meeting the study center’s criteria for starting sublingual immunotherapy, defined as either, |
| 3.a ≥4 AOM episodes in one year, or |
| 3.b Recurrent pharyngotonsillitis that meets the criteria for tonsillectomy in the Spanish ENT Society. |
| 4. Provision of a written informed consent to participate. |
| Exclusion criteria: |
| 1. Patients with a diagnosis of primary immunodeficiency, except for isolated IgA deficit. |
| 2. Patients under treatment with immunostimulant or immunosuppressant medications. |
| 3. Patients with metabolic, autoimmune, or chronic inflammatory/infectious diseases. |
| 4. Patients with obesity (body mass index greater than 30 kg/m^2^). |
| 5. Patients with syndromic diseases (Down, West, etc.). |
| 6. Patients with suspected or proven allergy to any of the components of the study product. |

Abbreviations: AOM, acute otitis media; ENT, ear, nose and throat; Ig, immunoglobulin; kg, kilogram; m, meter.

# Table S2: Solutions of the generalized linear mixed models

| **Parameter** |  | **Estimate ^a^** |  | **Std. error ^a^** |  | **P‑value** |
| --- | --- | --- | --- | --- | --- | --- |
| Poisson Distribution‑based model of the incidence of infect. episodes |  |  |  |  |  |  |
| Intercept |  | −1.950 |  | 0.305 |  | <0.001 |
| Visit (baseline vs. 6‑month) |  | 1.321 |  | 0.316 |  | <0.001 |
| Cohort (specific vs. auto-vaccines) |  | −0.666 |  | 0.300 |  | 0.032 |
| Baseline inter‑episode interval (≤1 month vs. >1 month) |  | 0.725 |  | 0.341 |  | 0.039 |
| Visit × cohort interaction |  | 0.481 |  | 0.312 |  | 0.130 |
| Visit × baseline inter‑episode interval interaction |  | −0.374 |  | 0.354 |  | 0.296 |
| Gamma Distribution‑based model of the severity of symptoms score | | | | | | |
| Intercept |  | −1.708 |  | 0.572 |  | 0.005 |
| Visit (baseline vs. 6‑month) |  | 3.396 |  | 0.642 |  | <0.001 |
| Cohort (specific vs. auto-vaccines) |  | 1.172 |  | 0.495 |  | 0.031 |
| Baseline inter‑episode interval (≤1 month vs. >1 month) |  | 0.929 |  | 0.437 |  | 0.050 |
| Subgroup (PT vs. AOM) |  | 0.848 |  | 0.373 |  | 0.037 |
| History of bronchitis (no vs. yes) |  | 1.135 |  | 0.472 |  | 0.029 |
| IgG_2_ (mg/dL) |  | −0.005 |  | 0.002 |  | 0.011 |
| Visit × cohort interaction |  | −1.064 |  | 0.553 |  | 0.073 |
| Visit × baseline inter‑episode interval interaction |  | −0.930 |  | 0.507 |  | 0.086 |
| Visit × subgroup interaction |  | −0.443 |  | 0.438 |  | 0.326 |
| Visit × history of bronchitis interaction |  | −1.251 |  | 0.521 |  | 0.029 |
| Visit × IgG_2_ interaction |  | 0.004 |  | 0.002 |  | 0.067 |
| Gamma Distribution‑based model of the school absenteeism score | | | | | | |
| Intercept |  | −3.529 |  | 1.145 |  | 0.004 |
| Visit (baseline vs. 6‑month) |  | 4.933 |  | 1.288 |  | 0.001 |
| Cohort (specific vs. auto-vaccines) |  | 4.790 |  | 0.971 |  | <0.001 |
| Baseline inter‑episode interval (≤1 month vs. >1 month) |  | 2.865 |  | 1.075 |  | 0.016 |
| Subgroup (PT vs. AOM) |  | 1.498 |  | 0.729 |  | 0.056 |
| IgG_2_ (mg/dL) |  | −0.012 |  | 0.003 |  | 0.003 |
| Visit × cohort interaction |  | −5.086 |  | 1.073 |  | <0.001 |
| Visit × baseline inter‑episode interval interaction |  | −2.920 |  | 1.186 |  | 0.025 |
| Visit × subgroup interaction |  | −1.164 |  | 0.855 |  | 0.192 |
| Visit × IgG_2_ interaction |  | 0.012 |  | 0.004 |  | 0.013 |

Abbreviations: infect., infectious; std., standard.

^a^ Values with logarithmic transformation.
